# Supplementary material for: A Likelihood Approach for Real-Time Calibration of Stochastic Compartmental Epidemic Models
Source: PLoS Comput Biol. 2017 Jan 17;13(1):e1005257. doi: 10.1371/journal.pcbi.1005257 (PMC5240920; doi:10.1371/journal.pcbi.1005257)
Supplement: S1 File — (TAR.GZ) [file pcbi.1005257.s014.tar.gz › HSPH_Online-SI-Revision/PF08/n10-extreme/n10-extreme_PF2_summary2.pdf]

peak −8	3w spec	3w cum	5w spec	5w cum	end	3w spec LOG	3w cum LOG	5w spec LOG	5w cum LOG	end LOG
mean	150.1	204.3	59.4	74.5	24.7	0.3	0.3	0.4	0.3	0.1
median	59.7	90.9	53.8	51.6	25.6	0.2	0.2	0.4	0.2	0.1
quantile .1	27.3	25.8	45.6	27.9	18.5	0.1	0.1	0.3	0.1	0.1
quantile .9	368.7	522.9	78.4	145.9	30.3	0.6	0.7	0.5	0.1	0.2
44	42	35	47	17						

peak −4	3w spec	3w cum	5w spec	5w cum	end	3w spec LOG	3w cum LOG	5w spec LOG	5w cum LOG	end LOG
mean	58.1	54.	51.7	54.6	21.4	0.4	0.4	0.4	0.4	0.1
median	57.4	53.6	52.1	54.	21.8	0.4	0.3	0.3	0.4	0.1
quantile .1	39.8	36.9	32.5	37.5	12.5	0.2	0.2	0.2	0.2	0.1
quantile .9	74.1	69.5	68.5	69.3	29.8	0.7	0.6	0.6	0.2	0.2
0	0	1	0	12						

peak	3w spec	3w cum	5w spec	5w cum	end	3w spec LOG	3w cum LOG	5w spec LOG	5w cum LOG	end LOG
mean	15.8	12.1	37.7	12.2	20.5	0.1	0.1	0.1	0.1	0.1
median	13.7	8.4	20.3	9.7	15.	0.1	0.	0.1	0.	0.1
quantile .1	5.3	2.6	6.	3.3	4.1	0.	0.	0.	0.	0.
quantile .9	29.5	25.7	77.7	22.4	40.8	0.1	0.1	0.2	0.	0.1
18	20	13	16	9						

peak +4	3w spec	3w cum	5w spec	5w cum	end	3w spec LOG	3w cum LOG	5w spec LOG	5w cum LOG	end LOG
mean	74.3	51.1	151.7	63.	89.4	0.2	0.2	0.3	0.2	0.2
median	45.7	29.8	77.	37.4	59.	0.2	0.1	0.2	0.1	0.2
quantile .1	14.8	8.	21.4	10.7	26.9	0.1	0.	0.1	0.	0.1
quantile .9	158.	101.3	271.2	128.5	175.3	0.4	0.3	0.6	0.	0.4
4	4	3	3	1						

peak +8	3w spec	3w cum	5w spec	5w cum	end	3w spec LOG	3w cum LOG	5w spec LOG	5w cum LOG	end LOG
mean	197.6	160.9	431.6	180.7	229.1	0.4	0.3	0.5	0.4	0.4
median	89.5	77.7	142.3	84.	107.2	0.3	0.2	0.4	0.3	0.3
quantile .1	40.7	30.	49.	32.	46.9	0.1	0.1	0.2	0.1	0.2
quantile .9	569.8	338.1	704.1	385.4	511.2	0.8	0.6	0.9	0.1	0.8
1	0	1	0	0	{}	{}	{}	{}	{}	{}
